# Antiphospholipid Antibodies Predict Progression of Abdominal Aortic Aneurysms

**DOI:** 10.1371/journal.pone.0099302

**Published:** 2014-06-30

**Authors:** Christina Duftner, Rüdiger Seiler, Christian Dejaco, Iris Chemelli-Steingruber, Harald Schennach, Werner Klotz, Michael Rieger, Manfred Herold, Jürgen Falkensammer, Gustav Fraedrich, Michael Schirmer

**Affiliations:** 1 Department of Internal Medicine, Clinic of Internal Medicine VI, Innsbruck Medical University, Innsbruck, Austria; 2 Department of Operative Medicine, Clinic of Vascular Surgery, Innsbruck Medical University, Innsbruck, Austria; 3 Department of Radiology, Innsbruck Medical University, Innsbruck, Austria; 4 Institute of Blood Transfusion and Immunology, Innsbruck Medical University, Innsbruck, Austria; 5 Department of Internal Medicine, General Hospital Kufstein, Kufstein, Austria; 6 Department of Rheumatology, Medical University Graz, Graz, Austria; 7 Department of Vascular and Endovascular Surgery, Wilhelminenhospital, Vienna, Austria; University of Leicester, United Kingdom

## Abstract

Antiphospholipid antibodies (aPLs) frequently occur in autoimmune and cardiovascular diseases and correlate with a worse clinical outcome. In the present study, we evaluated the association between antiphospholipid antibodies (aPLs), markers of inflammation, disease progression and the presence of an intra-aneurysmal thrombus in abdominal aortic aneurysm (AAA) patients.

APLs ELISAs were performed in frozen serum samples of 96 consecutive AAA patients and 48 healthy controls yielding positive test results in 13 patients (13.5%) and 3 controls (6.3%; n.s.). Nine of the 13 aPL-positive AAA patients underwent a second antibody testing >12 weeks apart revealing a positive result in 6 cases. APL-positive patients had increased levels of inflammatory markers compared to aPL-negative patients. Disease progression was defined as an increase of the AAA diameter >0.5 cm/year measured by sonography. Follow-up was performed in 69 patients identifying 41 (59.4%) patients with progressive disease. Performing multipredictor logistic regression analysis adjusting for classical AAA risk factors as confounders, the presence of aPLs at baseline revealed an odds ratio of 9.4 (95% CI 1.0–86.8, p = 0.049) to predict AAA progression. Fifty-five patients underwent a computed tomography in addition to ultrasound assessment indicating intra-aneurysmal thrombus formation in 82.3%. Median thrombus volume was 46.7 cm^3^ (1.9–377.5). AAA diameter correlated with the size of the intra-aneurysmal thrombus (corr_coeff_ = 0.721, p<0.001), however neither the presence nor the size of the intra-aneurysmal thrombus were related to the presence of aPLs.

In conclusion, the presence of aPLs is associated with elevated levels of inflammatory markers and is an independent predictor of progressive disease in AAA patients.

## Introduction

Antiphospholipid antibodies (aPL) are a group of heterogenous autoantibodies associated with spontaneous thrombus formation or pregnancy complications in patients with a disease recognized as antiphospholipid syndrome (APS) [1], [2]. APLs frequently occur in autoimmune and cardiovascular diseases, correlating with a worse clinical outcome of affected patients [3], [4]. The mechanisms leading to the evolvement of aPLs and their functional relevance in vascular diseases are still incompletely understood. Recent experiments indicated that aPLs inhibit the activated protein C pathway, lead to abnormalities in platelet function, up-regulate the tissue factor pathway and cause endothelial dysfunction collectively promoting aberrant thrombus formation and vascular damage [5].

Abdominal aortic aneurysms (AAA) are a common vascular disease with a prevalence of 3% in individuals aged 60 years or older. The pathogenesis of this disease appears to be complex, and immune-mediated mechanisms resulting in the activation of matrix metalloproteinases (MMP) with subsequent disruption of the orderly lamellar structure of the aortic media and tissue degradation play fundamental roles [6]. B-cells occur in the adventitia of AAAs [7] and pro-inflammatory CD4^+^ and CD8^+^ T-cells lacking the co-stimulatory molecule CD28 are enriched in peripheral blood and tissue specimens of AAA patients indicating the involvement of adaptive immune-responses in the pathogenesis of the disease [8].

It is well established that intra-aneurysmal laminated thrombi fill the lumina of AAAs to varying extents either covering the entire wall of AAAs or being eccentrically located, leaving part of the aneurysm wall exposed to blood flow. The growth of the intra-aneurysmal thrombus is associated with both, aneurysm progression and rupture [9], [10]. Intra-aneurysmal thrombi affect the underlying aortic vessel wall leading to chemotaxis of inflammatory cells, adsorption of plasma components and induction of apoptosis in smooth muscle cells [11]. Thus, the intra-aneurysmal thrombus functions as a site of protease release and activation, with subsequent degradation of the extracellular matrix [12].

Previous works reported the development of vascular aneurysms in patients with APS [13]. Given the known association of aPLs with immune-mediated and cardiovascular diseases as well as the evidence for immune-activation in AAA patients, we demonstrate in the Innsbruck AAA study cohort, that aPLs are associated with increased serological markers of inflammation and predict progressive disease.

## Materials and Methods

### Statement of the ethics committee

The ethics committee of the Innsbruck Medical University specifically approved this study during their session number 226/4.8, 289/5.6 (2618a), study number AM2249 on May 9, 2005 and most recently on May 5, 2010. Informed and written consent was obtained from each subject.

### Patients

All AAA patients included in this study are participants of a prospective investigation assessing the role of inflammatory biomarkers and immunocompetent cells in the pathogenesis of AAAs. Out of this AAA study cohort (n = 135) we randomly enrolled 96 AAA patients with an AAA diameter larger than 3 cm (Patients' characteristics and risk factors are summarized in Tables 1 and 2, respectively) as well as 48 healthy volunteers. We excluded neoplasms, chronic inflammatory diseases as well as infections by full medical history, clinical examination and routine blood tests in all subjects. Besides, none of the controls had a history of fetal loss, thrombotic events, thrombocytopenia, livedo reticularis or AAA.

**Table 1:** Patients' clinical characteristics.

	AAA patients	healthy controls	P-value
age [years]†	72 (46–85)	67.5 (50–96)	0.029
male sex, n (%), n = 96/n = 48	83 (86.5)	25 (52.1)	<0.001
AAA diameter [cm]†, n = 96	4.4 (3–8.7)	n. d.	
CD4^+^CD28^−^ T-cells [%]†, n = 92/n = 10	4.8 (0.1–33.8)	0.6 (0.3–2.5)	<0.001
CD8^+^CD28^−^ T-cells [%]†, n = 93/n = 10	39.9 (6.6–85.8)	26.3 (11.1–35.1)	0.001
Neopterin [ng/ml]†, n = 63/n = 18	7.8 (3.2–43.6)	7.3 (4.2–11.7)	n.s.
C-reactive protein [mg/dl]†, n = 89	1.0 (0.2–22)	n. d.	
ESR [mm/1^st^ hour]†, n = 46	10.0 (2–95)	n. d.	

n, number; AAA diameter (normal <2 cm); neopterin (normal <10 ng/ml); C-reactive protein (normal <0.7 mg/dl); ESR, erythrocyte sedimentation rate (normal <15 mm/1^st^ hour).

† median (range) as indicated for data with non-normal distribution and the Mann-Whitney U test was applied for comparison. The Chi-square test was used to test differences between proportions.

**Table 2:** Patients' clinical characteristics. Prevalence and data on traditional abdominal aortic aneurysm (AAA) risk factors.

AAA risk factors	
AAA diameter [cm]†, n = 96	4.4 (3–8.7)
volume thrombus [cm^3^]†, n = 55	46.7 (1.9–377.5)
male sex, n (%), n = 96	83 (86.5%)
smoking, n (%), n = 95	36 (37.9%)
hypertension, n (%), n = 95	55 (57.9%)
diabetes mellitus, n (%), n = 95	9 (9.5%)
hyperlipidaemia, n (%), n = 95	45 (47.4%)
coronary heart disease, n (%), n = 95	42 (44.2%)
peripheral arterial occlusive disease, n (%), n = 95	30 (31.6%)

n, number; AAA diameter (normal <2 cm);

† median (range) as indicated for data with non-normal distribution.

### Sonography and serial computed tomography volume measurements

Each AAA patient underwent ultrasound investigations of the abdominal aorta by an experienced vascular surgeon using a 2–5 MHz convex transducer (Toshiba Diagnostic Ultrasound System, Model SSA-660A). Sonographic determination of the aortic diameter was performed as part of clinical routine and disease progression was defined as an increase of the diameter by >0.5 cm per year [14].

A computed tomography (CT)-angiography was routinely performed in case of new diagnosis of AAA, difficulties with reliable measurement of the aortic diameter by sonography, suspected progression of AAA diameter above the threshold for surgical intervention and/or a follow-up of >5 years. Fifty-five (57.3%) AAA patients underwent a CT examination with a multi-slice CT-scanner (LightSpeed QX/i, GE Healthcare, Milwaukee, USA). After a native scan, depending on the patient's weight, up to 150 ml of a non-ionic contrast agent (Ultravist 370, Bayer HealthCare, Germany) were applied by means of an automatic injector using the SmartPrep software (GE Healthcare) with a flow rate of 4 ml/s. CT-scans were performed during the early arterial and the delayed phase with a slice thickness of 2.5 mm and 5 mm, respectively. Using the axial source images, multiplanar reformations, volume rendering reconstruction and maximum intensity projections as well as volumetric measurements were performed on a postprocessing system (Advantage Windows Ultra Spark 10 Work Station 3.1, Sun Microsystems, Mountain View, CA, USA). Volumes were assessed using the summation area technique, outlining the volume of interest on each CT with the cursor. The postprocessing system automatically determined boundaries around a class of similar voxel intensity values, and the software calculated the volume of all marked areas.

The following 3 volumes of interest were defined semi-automatically: the contrast-filled intra-aneurysm vascular lumen, the total aneurysm volume of the aneurysm sac and the volume of the aneurysm thrombus. The total aneurysm volume included the volume of the aneurysm thrombus and lumen. The volume of the aneurysm thrombus was calculated by subtracting the intra-aneurysm vascular channel from the total aneurysm volume [15]. Volume analysis started at the level of the coeliac trunc and ended at the aortic bifurcation.

### Antiphospholipid antibody tests and diagnosis of antiphospholipid syndrome

ELISAs were performed according to the manufacturers' instructions to screen for aPLs (Anti-Phospholipin Screen IgG/M Kit ORG 529, Orgentec, Mainz, Germany), for antibodies directed against cardiolipin (CL) (Aeskulab – Aeskulisa Cardiolipin-GM 7204, Germany) and beta-2-Glycoprotein I (β2GPI) (708665 QUANTA Lite B2 GPI IgG, INOVA, San Diego, CA and β2GPI IgG/M Kit ORG 521, Orgentec, Mainz, Germany). The cut-off levels used were 15 GPL/ml and MPL/ml for IgG and IgM antibodies directed against cardiolipin, respectively, 20 SGU/ml for β2GPI IgG and 5 U/ml for β2GPI IgM antibodies as well as 10 U/ml for IgG and IgM aPL screening antibodies. Medium/high titers of aPLs were defined in accordance with previous publications as follows: CL antibody of IgG and/or IgM isotype in serum >40 GPL or MPL, or >than the 99^th^ percentile and β2GPI antibody of IgG and/or IgM isotype in serum or plasma with a titer >99^th^ percentile [2].

### Inflammatory parameters and three color immunofluorescence flow cytometry

For neopterin measurement the automated version of a commercially available ELISA (ELItest Neopterin, BRAHMS, Berlin, Germany) was used according to the manufacturer's instructions (interassay coefficient of variation: 6.54%, limits of detection without dilution: between 2 and 50 nmol/L). Erythrocyte sedimentation rate (ESR) was determined by the Westergren method.

Percentages of CD28^−^ T-cells out of the CD3^+^CD4^+^ and CD3^+^CD8^+^ peripheral blood mononuclear cells (PBMCs) as markers for chronic inflammation were determined by FACS surface staining using fluorescein isothiocyanate conjugated anti-CD4, anti-CD8, phycoerythrin-conjugated anti-CD28 and peridinin chlorophyll protein-conjugated anti-CD3 monoclonal antibodies (all from Becton Dickinson, San Diego, CA, USA) as previously described [8]. Cells were analysed on a FACS-Calibur flow cytometer (Becton Dickinson) and data calculated using WinMDI software (Version 2.8, Joseph Trotter, Scripps Research Institute, La Jolla, CA, USA).

### Statistics

Statistical analyses were performed using the SPSS program, version 21.0 (Chicago, IL, USA). The Kolmogorov-Smirnov test was used to test for normal distribution of metric parameters. Normally distributed values are expressed as mean ± standard deviation (SD) and were compared by the Student's t test. In case of non-normally distributed data the median and range are given and the Mann-Whitney-U test was applied. Correlations were assessed by the Spearman rank correlation test. Proportions were calculated by the Chi-square or Fisher's exact tests as appropriate. We performed multivariate logistic regression analysis (maximum likelihood method) to investigate the association between aPLs and AAA disease progression adjusting for possible confounders (age, sex, history of smoking, arterial hypertension, hyperlipidaemia, diabetes mellitus, coronary heart disease, peripheral arterial occlusive disease and AAA diameter). A stepwise backward regression model was conducted excluding parameters failing the pre-specified significance level of 0.1. Goodness of fit was tested using Hosmer-Lemeshow statistics and sensitivity analyses were assessed excluding influential points as well as cases with large Cook and/or DFBETA values. p<0.05 was considered as significant.

## Results

### Baseline examinations of antiphospholipid antibodies

Results of aPL testing including antibody subtypes are detailed in Table 3. The prevalence of aPLs seemed to be higher in AAA patients (13.5%) than in healthy controls (6.3%), however, the difference did not reach statistical difference (p = 0.189, using the Chi-square test). The presence of aPLs was not related to sex, neither in AAA patients nor in healthy controls.

**Table 3:** Baseline prevalence of antiphospholipid antibodies (aPL) in consecutive abdominal aortic aneurysm (AAA) patients and healthy controls.

	AAA patients	healthy controls	P-value
**aPL positivity**	13/96 (13.5%)	3/48 (6.3%)	n.s.
aPL subsets, n (%*)	1 (7.7%) β2GPI IgG+		
	5 (38.5%) CL IgG+		
	2 (15.4%) CL & β2GPI IgG+		
	1 (7.7%) β2GPI IgM+		
	1 (7.7%) CL IgM+	2 (66.7%) CL IgM+	
	3 (23.1%) CL & β2GPI IgM+	1 (33.3%) CL & β2GPI IgM+	

β2GPI, β2 glycoprotein I antibodies; CL, cardiolipin antibodies.

*out of aPL-positive patients.

Proportions were compared using the Chi-square test.

Medium or high serum titers of antibodies directed against CL or β2GPI were found in 3 (3%) AAA patients and in 1 (2.1%, p>0.2) healthy control [2]. Nine of the 13 AAA patients with a positive aPL result underwent a second blood test >12 weeks later. Six (66.7%) patients showed persistent aPL antibodies (Table 4).

**Table 4:** Prevalence of persistent antiphospholipid antibodies (aPL) defined as 2 positive test results >12 weeks apart in abdominal aortic aneurysm (AAA) patients.

**aPL double-positivity [%]**	6/9 (66.7%)
aPL subsets, n (%*)	3 (50%) CL IgG+
	1 (16.7%) CL & β2GPI IgG+
	1 (16.7%) CL IgM+
	1 (16.7%) CL & β2GPI IgM+

Nine of 13 patients with a positive test result underwent a second antibody testing.

β2GPI, β2 glycoprotein I antibodies; CL, cardiolipin antibodies.

*out of aPL-positive patients.

### Antiphospholipid antibodies and AAA disease progression

Clinical and sonographic follow-up examinations were performed in 69 patients (including 10/13 aPL positive AAA patients) over a median of 46.5 months (5–72). Of these 69 patients, 28 (40.6%) had stable and 41 (59.4%) progressive disease as measured by ultrasound and defined as an increase of more than 0.5 cm/year [9]. Those AAA patients with progressive disease revealed larger AAA diameters [4.4 cm (3.2–7)] compared to patients with stable disease [3.9 cm (3–6.5), p = 0.037 performing the Mann-Whitney-U test].

AAA patients with a positive aPLs test result showed disease progression more frequently than aPL-negative patients [9/10 patients (90%) versus 32/59 patients (54.2%), p = 0.041 according to the Fisher's exact test].

We performed step-wise backward logistic regression analysis with AAA progression as the dependent variable, aPLs as the predictor of primary interest and traditional risk factors for AAA progression (age, sex, history of smoking, arterial hypertension, hyperlipidaemia, diabetes mellitus, coronary heart disease, peripheral arterial occlusive disease and AAA diameter) as possible confounders. All parameters failed the pre-specified significance level of 0.1 except for aPLs (OR 9.4, 95% CI 1.0–86.8, p = 0.049), AAA diameter (OR 3.3, 95% CI 1.5–7.4, p = 0.004) and peripheral arterial occlusive disease (OR 4.1, 95% CI 1.2–14.3, p = 0.028). The final inclusive model considering all traditional risk factors revealed an OR of 9.6 (95% CI 1.0–94.1, p = 0.053) for aPLs to predict AAA progression. aPLs were neither related to the AAA diameter nor to peripheral artery occlusive disease.

### Increased levels of inflammatory markers in patients with antiphospholipid antibodies

**Figure 1:**
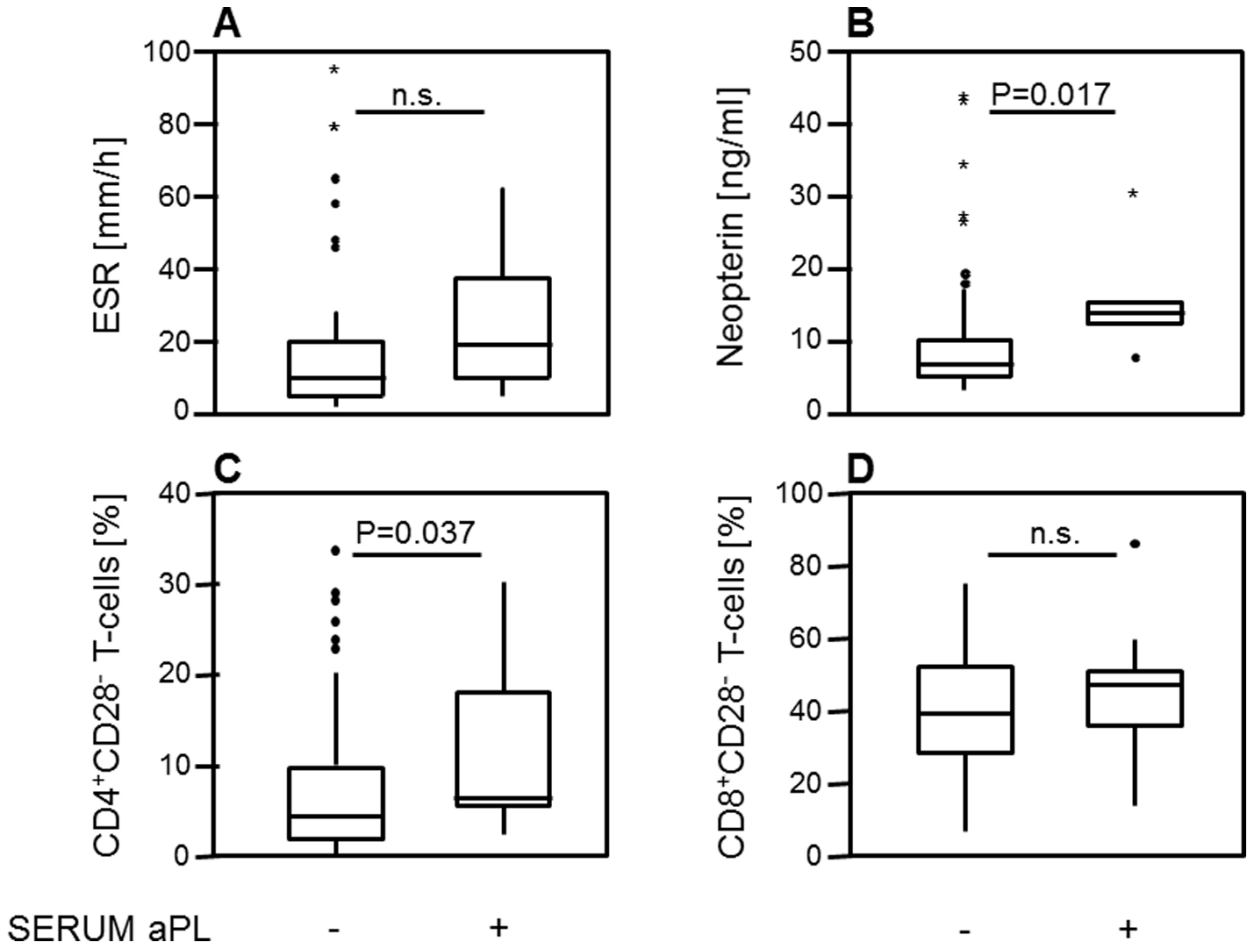
Levels of inflammatory markers in antiphospholipid antibodies (aPL)-negative and aPL-positive abdominal aortic aneurysm (AAA) patients. (**A**) Erythrocyte sedimentation rate (ESR) was not elevated in aPL-positive AAA patients. (**B**) Serum levels of neopterin were increased in aPL-positive AAA patients compared to aPL-negative AAA patients (p = 0.017). (**C**) Peripheral levels of CD4^+^CD28^−^, but not of (**D**) CD8^+^CD28^−^ T-cells as markers of chronic inflammation were increased in aPL-positive AAA patients compared to aPL-negative AAA patients (p = 0.037). Whiskers box plots show 50% of cases within the boxes and all data excluding mavericks between the end-points of the whiskers (lines). P-values <0.05 were considered as significant (using the Mann-Whitney U test).

Baseline aPL positive AAA patients had higher serum levels of neopterin [14 ng/ml (7.7–30.4) versus 6.8 ng/ml (3.2–43.6), p = 0.017] as well as increased prevalences of circulating CD4^+^CD28^−^ T-cells [6.4% (2.3–30.2) versus 4.4% (0.1–33.8), p = 0.037] compared to aPL negative patients (see Figures 1B and 1C, respectively). No differences were found between aPL positive and aPL negative AAA patients regarding ESR and peripheral levels of CD8^+^CD28^−^ T-cells (Figures 1A and D, respectively).

### Antiphospholipid antibodies and intra-aneurysmal thrombus formation

**Figure 2:**
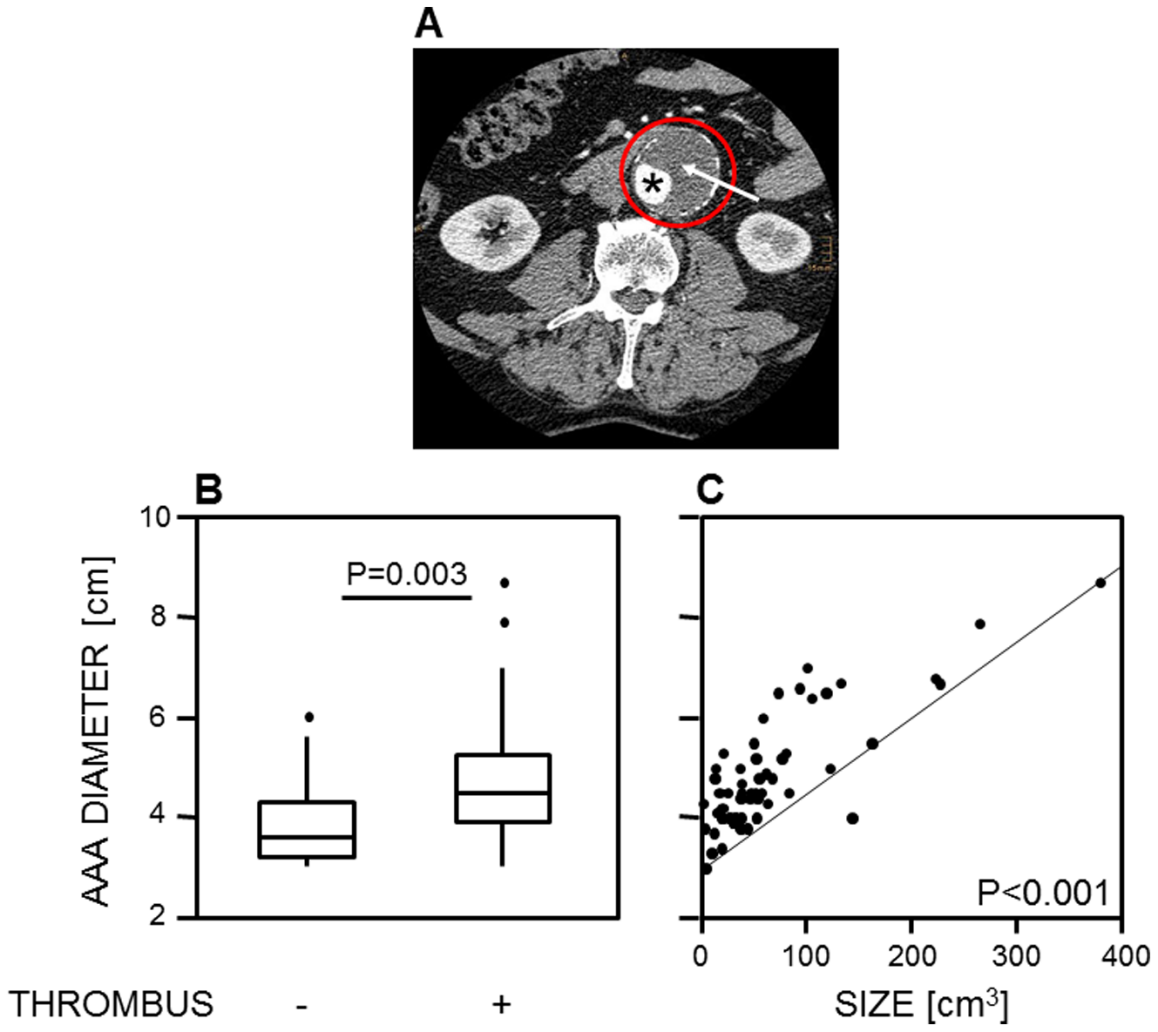
An intra-aneurysmal thrombus was present in 82.3% of abdominal aortic aneurysm (AAA) patients. (**A**) Example of an intra-aneurysmal thrombus affecting ≥50% of the AAA lumen (red circle), which was calculated to be 194.5 cm^3^ by serial computed tomography volumetry. (**B**) The presence of an intra-aneurysmal thrombus was associated with larger AAA diameters as assessed by the Mann-Whitney U test. Whiskers box plots show 50% of cases within the boxes and all data excluding mavericks between the end-points of the whiskers (lines). (**C**) AAA diameters were associated with the volume of the intra-aneurysmal thrombus ranging from 1.9 to 377.5 cm^3^, evaluated by the Spearman-Rho test (corr_coeff_ = 0.721, p<0.001). P-values <0.05 were considered as significant.

Of 96 AAA patients, 79 (82.3%) presented with an intra-aneurysmal thrombus (example given in Figure 2A). The median volume of the thrombus measured by CT was 46.7 cm^3^ (1.9–377.5). AAA patients presenting with an intra-aneurysmal thrombus revealed larger AAA diameters [4.5 cm (3–8.7)] compared to those patients without intra-aneurysmal thrombus [3.6 (3–6) (p = 0.003, according to the Mann-Whitney-U test)] as outlined in Figure 2B. The AAA diameter correlated with the size of the intra-aneurysmal thrombus (corr_coeff_ = 0.721, p<0.001, Figure 2C). The presence of an intra-aneurysmal thrombus, however, was not related to AAA progression as determined by Fisher's exact test.

An intra-aneurysmal thrombus formation was found in 11/13 (84.6%) and 68/83 (81.9%, n.s.) patients with a positive or negative aPL test result at baseline, respectively. Out of the 11 aPL positive patients with thrombus, 5 (45.5%) had a second positive test result, and 8/11 (72.7%) (including 4/5 patients with persistent aPLs) presented with progressive disease. aPLs were not associated with the thrombus volume.

## Discussion

This study revealed higher serological signs of inflammation in aPL-positive compared to aPL-negative AAA patients and identified aPLs as a predictor of progressive disease.

All except one aPL-positive patients suffered from progressive disease (90%), whereas only 54.2% of aPL-negative patients showed AAA progression. Multivariate logistic regression analysis revealed aPLs, baseline AAA diameter as well as peripheral arterial occlusive disease as predictors for AAA progression, whereas other traditional risk factors failed the predefined significance level. The most likely explanation for this result is the limited power of our regression analysis to detect weaker associations between predictors and the dependent variable given the relatively low number of cases and the high number of items included. However, an inclusive model forcing all traditional risk factors into the analysis model (regardless of their significance level) did not alter the association between aPLs and AAA progression thus proving the validity of our step-wise model.

An association between aPLs and poor outcome of immune-mediated and cardiovascular disease is well established: In systemic lupus erythematosus patients for example, the aPL IgG mediated production of tissue factor and pro-inflammatory cytokines by peripheral blood mononuclear cells contributed to arteriosclerosis [16]. Besides, aPL positive systemic lupus erythematosus patients had an increased mortality rate [17] and patients with giant cell arteritis and aCL antibodies suffered from recurrent flares [18]. Although AAA has been considered as a degenerative disease for many years, recent work suggests an important role of immune-mediation for the pathogenesis of AAAs. In line with this new concept, a case series on 4 APS patients with coexisting AAA reported an early and rapid progression of disease with an AAA-growth rate of >1.5 cm within 5 months in one of them [13]. Also in patients with cardiovascular diseases, aPLs predicted first myocardial infarction [19] or recurrent stroke [20].

The prevalence of aPLs in 13.5% of AAA patients was consistent with the prevalence of these antibodies in other immune-mediated diseases such as systemic lupus erythematosus (12–30% of patients are positive for CL antibodies), early rheumatoid arthritis (15.7%) and giant cell arteritis (25.5%) [21]–[24]. In patients with primary and secondary APS, particularly in cases with underlying vasculitis, an association between aPLs and the development of vascular aneurysms was previously reported [13], [25]–[29]. In young healthy subjects aPLs were found in 1–5% of cases [21], and with advancing age these autoantibodies are more frequently observed as indicated by a prevalence of 6.3% in our healthy elderly cohort [21], [30].

From the pathophysiological perspective, our findings further support the concept of immune-mediated processes contributing to the development and progression of AAA disease. We previously demonstrated that early aged pro-inflammatory T-cells are present in the peripheral blood and tissue specimens of AAA patients [8], and the present study indicates an involvement of B-cells via abnormal production of aPLs in the pathogenesis of the disease as well. The effect of aPLs on AAA progression possibly results from aPL-mediated enhanced MMP-9 activity [31] and/or aPL-induced acceleration of elastin degradation with consecutive remodelling of the arterial wall [32].

As previously reported, we confirmed an association between AAA diameter and the volume of the intra-aneurysmal thrombus [33]–[35]. The role of the intra-aneurysmal thrombus for AAA progression, however, is still controversial: several studies demonstrated that the size of an intra-aneurysmal thrombus is related to AAA progression and/or rupture [12], [36]–[38]. The intra-aneurysmal thrombus may function as a site of protease release and activation [12]. Besides, it may promote oxygen derived free radical development thus contributing to the destruction of the media and leading to AAA progression [38]. In contrast, in our cohort we found no association between thrombus formation and aneurysm growth. This finding is in line with others showing that a thrombus may even reduce shear-stress imposed on the AAA wall [39]–[41]. In summary, the factors determining whether an intra-aneurysmal thrombus increases the risk for AAA progression or protects against deterioration of the disease are still unknown [35].

The limitations of our study are the following: First, we cannot exclude a type II error due to the small sample size of aPL-positive AAA patients (post-hoc power calculation: 28.5%), and the differences between AAA patients and controls concerning age and sex reduced the power to detect a higher aPL prevalence in the former compared to the latter group. The focus of this study, however, was not a prevalence analysis; we rather aimed to examine the possible value of aPLs as a marker of immune activation and as a risk factor for AAA progression. Second, we determined CL- and β2GPI-antibodies by an aPL screen kit, but did not investigate the lupus anticoagulant, which is required for complete evaluation of APS [2]. Third, none of our controls had a history of AAA, however, we did not perform an ultrasound examination of the abdominal aorta to exclude this disease. Fourth, data on current medication of AAA patients were not available and the effects of drugs with pleiotropic effects on the immune system and/or the aortic wall such as statins, angiotensin converting enzyme and angiotensin receptor inhibitors could not be evaluated [42], [43].

In conclusion, aPLs occurring in AAA patients are associated with increased serological markers of inflammation and predicted progressive disease. These findings further support the concept of possibly underlying immune-mechanisms in AAA development. No association was observed between aPLs and the presence or size of an intra-aneurysmal thrombus.
